# Environmental non-governmental organizations and global environmental discourse

**DOI:** 10.1371/journal.pone.0232945

**Published:** 2020-05-27

**Authors:** Stefan Partelow, Klara Johanna Winkler, Gregory M. Thaler

**Affiliations:** 1 Department of Social Sciences, Leibniz Centre for Tropical Marine Research (ZMT), Bremen, Germany; 2 Department of Natural Resource Sciences, McGill University, Ste-Anne-de-Bellevue, Canada; 3 Department of International Affairs, University of Georgia, Athens, Georgia, United States of America; University of Maryland Baltimore County, UNITED STATES

## Abstract

Environmental non-governmental organizations (ENGOs) exist worldwide, and since the 1980s they have increasingly influenced global environmental politics and environmental discourse. We analyze an original dataset of 679 ENGOs participating in global environmental conventions in the mid-2010s, and we apply quantitative content analysis to ENGO mission statements to produce an inductive typology of global environmental discourse. Discourse categories are combined with ENGO attribute data to visualize the political topology of this globally-networked ENGO sector. Our results confirm some common assertions and provide new insights. ENGOs are more diverse than conventionally recognized. Quantitative evidence confirms strong North-South disparities in human and financial resources. Four primary discourses are identified: Environmental Management, Climate Politics, Environmental Justice, and Ecological Modernization. We compare our typology to existing literature, where Climate Politics and Environmental Justice are under-appreciated, and we discuss ways to expand on the data and methods of this study. Synoptic empirical ENGO research is essential to accurately understanding the ENGO sector and global environmental politics.

## Introduction

The increasing number and importance of non-governmental organizations (NGOs) has been a central feature of international politics since the 1980s [1,2]. NGOs are especially prominent in environmental politics, where the expansion of environmental NGOs (ENGOs) has both driven and responded to shifts from state-centered environmental regulation to polycentric ‘governance’ configurations that include governments, ENGOs, intergovernmental organizations, corporations, and social movements [3–6]. ENGOs now have substantial impacts on people and their environments, both on the ground through project implementation [7–11] and through influence on policy from the local to international levels [3,12–15].

From the 1950s to 1990s, ENGOs comprised a growing proportion of the international NGO sector [12] (p.11). The 1992 Rio Earth Summit (also known as the United Nations Conference on Environment and Development (UNCED), or Earth Summit) was a catalyst for new ENGO formation and the development of a global environmental governance regime [3]. The resulting Rio Conventions—the United Nations Framework Convention on Climate Change (UNFCCC), United Nations Convention to Combat Desertification (UNCCD), and Convention on Biological Diversity (CBD)—have served as foci for contesting and coordinating environmental policy among intergovernmental organizations, governments, NGOs, and other actors. ENGOs are linchpins in the global environmental governance regime, linking the local with the global and the biophysical with the political [3]. ENGOs thus also help structure the discursive field within which environmental ‘problems’ are defined and policy responses constructed [16–18].

A comprehensive empirical overview of the global ENGO sector is lacking. Most existing studies are characterized by broad generalizations [19–21], or by single-case or small-*N* comparative research [8,18,22–25]. The few comprehensive surveys of the ENGO sector include the 1998 *Global Environmental Organizations Survey* (GEOS) of 248 ENGOs in 59 countries, which revealed that ENGO resources and ideology influence patterns of political action [26] and described North-South resource transfers between ENGOs across substantial power and value asymmetries [27]. Brockington and Scholfield collected data on 281 conservation NGOs working in sub-Saharan Africa in 2004–2006, and found an unequal distribution of financial resources in the sector and uneven geographies of conservation expenditure [28,29]. In another regional study, Hoffman’s [30] social network analysis of 69 US-registered ENGOs further demonstrated benefits of synoptic research, identifying five distinct roles played by ENGOs in corporate networks. GEOS is now outdated, however, and other analyses have been of more limited geographical scope.

Crucially, NGOs participating in environmental politics are not limited to “green-chip ENGOs” (p. 516) [19], household names like WWF and Greenpeace. Rather, ENGOs include diverse scientific, humanitarian, and special-interest groups ranging from the African Centre for Technology Studies (Kenya) to the Norwegian Refugee Council (Norway). Existing ENGO studies are biased toward conventional environmental conservation and advocacy organizations, however (e.g., 15). Current knowledge of ENGOs is thus partial and incomplete.

Meanwhile, prior studies of environmental discourse suffer from lack of rigorous methodologies for creating discourse typologies. A discourse is “a shared way of apprehending the world” (p. 8) [31], comprising a system of ideas, definitions, and values that structure understanding and action. Surveys of ENGO discourse have been limited to green-chip organizations [17,32], and the broader environmental discourse literature derives typologies either deductively from rhetorical theory [18,33] or inductively without rigorous methodological specification [17,31,34–36]. Deductive typologies rely on Ogden and Richards’ [37] semiotic triangle, and identify a scientific discourse of ‘nature as object,’ a regulatory discourse of ‘nature as resource,’ and a poetic discourse of ‘nature as spirit’ [32,33,38]. These typologies are useful heuristics but do not derive from actually-existing discourses. Meanwhile, inductive typologies have generally left their methodologies implicit or underspecified [17,31,34–36], though Sandbrook et al. [39] identified three ‘dimensions of conservation thinking’ based on factor analysis of a survey of conservationists. We find no typology derived from systematic analysis of existing discourse.

We address these gaps by analyzing an original worldwide dataset of 679 ENGOs accredited to the Rio Conventions. This population comprises a subset of the ENGO universe. Focusing on ENGOs participating in the Rio Conventions [40] allows us to survey a population networked through major global environmental governance forums. We describe the geographical and organizational diversity of this globally-networked ENGO population, and we use quantitative content analysis of ENGO mission statements to produce an inductive typology of environmental discourse. Combining our discourse categories with ENGO attribute data reveals the political topology of this portion of the ENGO sector. We compare our analysis with existing environmental discourse typologies and discuss implications of our findings for understanding ENGOs and environmental discourse. While our dataset captures a core of globally-networked ENGOs, it does not represent the full diversity of the ENGO universe, and we propose directions for future research to expand on our approach and develop a more complete empirical understanding of the global ENGO sector.

## Materials and methods

Additional methodological details are provided in the Methods, in S1 Appendix.

### Research design

We analyze an original ENGO survey, which we use to create a typology of ENGO discourse and to describe key spatial and structural characteristics of a globally-networked ENGO population. We defined our target population of ENGOs as those accredited to the UNFCCC [41] (United Nations Framework Convention on Climate Change), UNCCD [42] (United Nations Convention to Combat Desertification), and CBD [43] (United Nations Convention on Biological Diversity). This population admits non-profit organizations from anywhere in the world that applied for and were granted observer status with at least one Rio Convention, regardless of whether those groups are conventionally considered ENGOs. The population excludes ENGOs that do not have the capacity or interest to participate in Rio Convention meetings. This empirical selection strategy reduces selection bias due to researchers’ preconceived definitions of ENGOs, and produces a population comprising organizations that are critical to the development of global environmental policy discourse. We are interested not in groups categorized as ENGOs *a priori*, but rather in the population of organizations that participate as ENGOs at global conferences. ‘Global environmental discourse’ in this study refers specifically to the discourse of ENGOs that participate in global environmental governance forums (i.e., the Rio Conventions). Our dataset comprises a snapshot of this ENGO sector at the time of research in 2014–2016.

Many studies of international NGOs define their population using *The Yearbook of International Organizations* [44], including in environmental research (e.g., [45,46]). Our alternative strategy allows ENGOs to self-select into the population and does not limit ENGOs to explicitly international organizations. As Dodds [47] notes, at the UN, the term NGO means ‘not government,’ but groups may be more or less closely tied to governments. We do not evaluate these ties, but rather provide an overview of the ENGO sector as defined in practice at the Rio Conventions. Our selection strategy assumes that those ENGOs accredited to the Rio Conventions are the most important ENGOs for the construction of global environmental governance and discourse. On the importance of global environmental conferences to global environmental governance, see Haas [48] and Corson et al [49].

We describe this globally-networked ENGO sector based on organizational attributes of nationality, founding year, number of employees, and annual budget. We also analyze ENGO discourse based on organizations’ mission statements. Mission statements are crystallizations of organizational discourse and goals [50]. While they may not directly predict organizational identities, practices, and outcomes [22,51,52], mission statements represent meaningful and intentional communication that both reflects and structures organizational practice [26,53]. Mission statements are especially important in the nonprofit sector for communicating organizational purpose, and they are used by government and private regulators as indicators of nonprofit accountability to the public interest [54]. As such, mission statements provide key data for large-*N* studies of organizational discourse, including in the environmental field [32,51]. We analyze ENGO discourses inductively using quantitative content analysis to derive a discourse typology. Last, we map the topology of the sector by combining the discourse typology with ENGO organizational attributes, bringing together environmental ideology and structural power.

### Data collection and formatting

The three Rio Conventions structure civil society participation differently. The UNFCCC Secretariat lists civil society organizations (CSOs) admitted as observers to the UNFCCC process. Admitted groups may register for meetings and self-select into constituencies that mirror ‘major groups’ established in Agenda 21. ENGOs comprise one of these major groups. We downloaded the list in May 2016 and included the 745 organizations in the UNFCCC ENGO constituency in our population. The UNCCD Secretariat maintains a similar list of accredited CSOs, but does not distinguish these CSOs by major groups. We obtained the list of UNCCD organizations accredited as of November 2015 and manually classified CSOs accredited to UNCCD as ‘ENGO’ or ‘other’ based on the CSO’s name and official website, yielding 295 ENGOs. The CBD does not maintain a general list of accredited CSOs; rather, organizations must apply for observer status for specific CBD meetings. CBD observers are categorized according to ‘major group’ designations. We obtained the list of observer organizations that participated in the 12^th^ CBD Conference of the Parties (COP) in South Korea in October 2014 and included the 108 organizations in the NGO category in our population. After accounting for ENGOs participating in multiple conventions, our final population consists of 978 individual ENGOs.

We collected attribute data (nationality, founding year, employees, and budget) and mission statements for these 978 ENGOs from their official websites (Table 1). Where we could not find an official website or English-language mission statement, we contacted the organization via email. The final dataset comprises the sample of 679 ENGOs for which we obtained English-language mission statements, representing 69.4% of the ENGO population. This sample slightly over-represents European (+3.4%) and Northern American (+5.5%) ENGOs and under-represents African (-6.3%) and Latin American and the Caribbean (LAC) (-6.2%) groups relative to the general population (S1 Table in S1 Appendix). We speculate that some African and LAC ENGOs with few resources may work primarily in French, Spanish, or Portuguese but not English, and may therefore be less likely to publish an English-language mission statement or respond to an English-language email. Our African sample skews towards Anglophone countries, and we suspect our African and LAC samples both skew towards organizations with greater human and financial capacity (cf. [55]). For the ENGO population participating in Rio Convention meetings, where English is often the primary working language [56], the bias produced by our English-language methodology does not appear extreme, and regional geographical distribution of our sample does not deviate more than 6.3% from the population distribution.

**Table 1:** Summary of collected data for sample (*N* = 679). We use United Nations M49 Standard Geographic Regions, available: https://unstats.un.org/unsd/methodology/m49/.

Category	Definition	Data type	Data range	*N*
ENGO name	Official name of ENGO	Text	--	679
Country	Country where registered or main office is located	Categorical	89 countries	679
Region	UN Geographic Region	Categorical	Asia; Africa; Oceania; Europe; Northern America; Latin America and the Caribbean (LAC)	679
Mission statement	Mission statement of ENGO	Text	--	679
UN Convention	Convention where accredited as observer	Categorical	UNFCCC; UNCCD; CBD	679
Year founded	Stated year ENGO established	Ordinal	1826–2014	601
Employees	Number of full-time employees	Numerical	0–17,319	432
Budget	Most recent total annual income in 2016 USD	Numerical	7,000–2,180,556,000	336

Additionally, we calculated a novel ENGO structural power index (*N* = 276). Taking human and financial resources as components of organizational power, budget and employee data were normalized and averaged together to produce a single index value representing the structural power of each ENGO (see S1 Appendix, Methods). We divided the index into quartiles to identify ENGOs with high, medium-high, medium-low, and low structural power.

### Content analysis

We inductively derived a typology of ENGO discourse through quantitative content analysis of ENGO mission statements using MaxQDA [57]. In the first-stage analysis, we generated a list of the 100 most frequent single words and a list of the 100 most frequent meaningful word combinations of two or three words (e.g., climate change, natural resource management) across all mission statements. Part of speech permutations (e.g., *environmental* and *environment*) are lemmatized to the most commonly occurring permutation and treated as a single word. These lemmatized words are used in reporting content analysis results, though in some cases we report parts of speech different from the lemmatized root to facilitate legibility. We identified 11 categories across the most frequently occurring terms using inductive coding. The lemmatization and coding processes are detailed in the Methods, S1 Appendix. The full list of lemmatized roots and frequencies is included in attached data.

In the second-stage analysis, we generated a binary dataset indicating occurrence of each of the most frequent words and word combinations for all mission statements. We conducted a principal component analysis (PCA) of the binary dataset of word occurrence to identify clusters of terminology that explain variation in ENGO discourse. Our goals with PCA were to “extract the most important information… [to] simplify the description of the data set; and analyze the structure of the observations,” [58] (p. 434). PCA is a common technique for identifying discourses in content analysis [59,60], including in Q-Method, which has been applied extensively in environmental research [61,62].

Different approaches exist for obtaining and plotting principal component (PC) scores in PCA [63]. Our analysis tailors to the nature of our data (which have both quantitative values and qualitative meaning) and the large number of variables (i.e., words in the raw data). We use a scree plot test [64,65] to identify two principal components (PCs) as most influential. Reducing the number of variables through PC loadings enables us to identify meaningful patterns based on terms that explain the most variation in the dataset. Similar approaches have recently been applied in the environmental sustainability literature [66–68]. The two most influential PCs (each containing 88 terms) are interpreted as spectrums that explain variation in the words within each mission statement, where terms loading most strongly positive on a PC are more likely to co-occur in a mission statement with other positive terms, and less likely to co-occur with terms that load strongly negative. Two PCs thus produce four clusters of terminology (two from each PC), differentiating four environmental discourses. PC1+ comprises 46 words, PC1- comprises 42 words, PC2+ comprises 69 words, and PC2- comprises 19 words. We use the top 15 words from each cluster as indicator terminology for naming each discourse.

To map the position of individual ENGOs within the discursive field described by this typology, we present a methodological addition that imagines the two PCs as X and Y axes of a discursive plane. We assigned a value to each mission statement on each axis by coding for the presence of the 15 indicator words for each discourse. A mission statement receives +1 for each indicator word it contains from the positive end of the PC and -1 for each indicator word it contains from the negative end of the PC. A mission statement containing all words from the positive end and no words from the negative end would receive a score of +15, indicating the strong and exclusive usage of that discourse. A statement containing all indicator words from both discourses would receive a 0, as it would participate strongly in both discourses, but is not a distinct representative of either. This classification method reflects the logic of PCA, namely, that discourses are defined in contradistinction to each other, insofar as they explain variation, and are known through actors that employ one set of concepts and not another. The two PC scores are plotted as (X,Y) values to show the distribution of ENGOs across the discursive field.

## Results

The largest proportion of ENGOs in our dataset is admitted to UNFCCC (S2 Fig in S1 Appendix). In Africa, Asia, Europe, and Latin America and the Caribbean (LAC), less than a third of ENGOs are observers at UNCCD or CBD. Nearly all Northern American and Oceanian ENGOs are admitted to UNFCCC, with proportionally little presence at other conventions (S5 Fig in S1 Appendix).

### Geographical and historical trends

**Fig 1:**
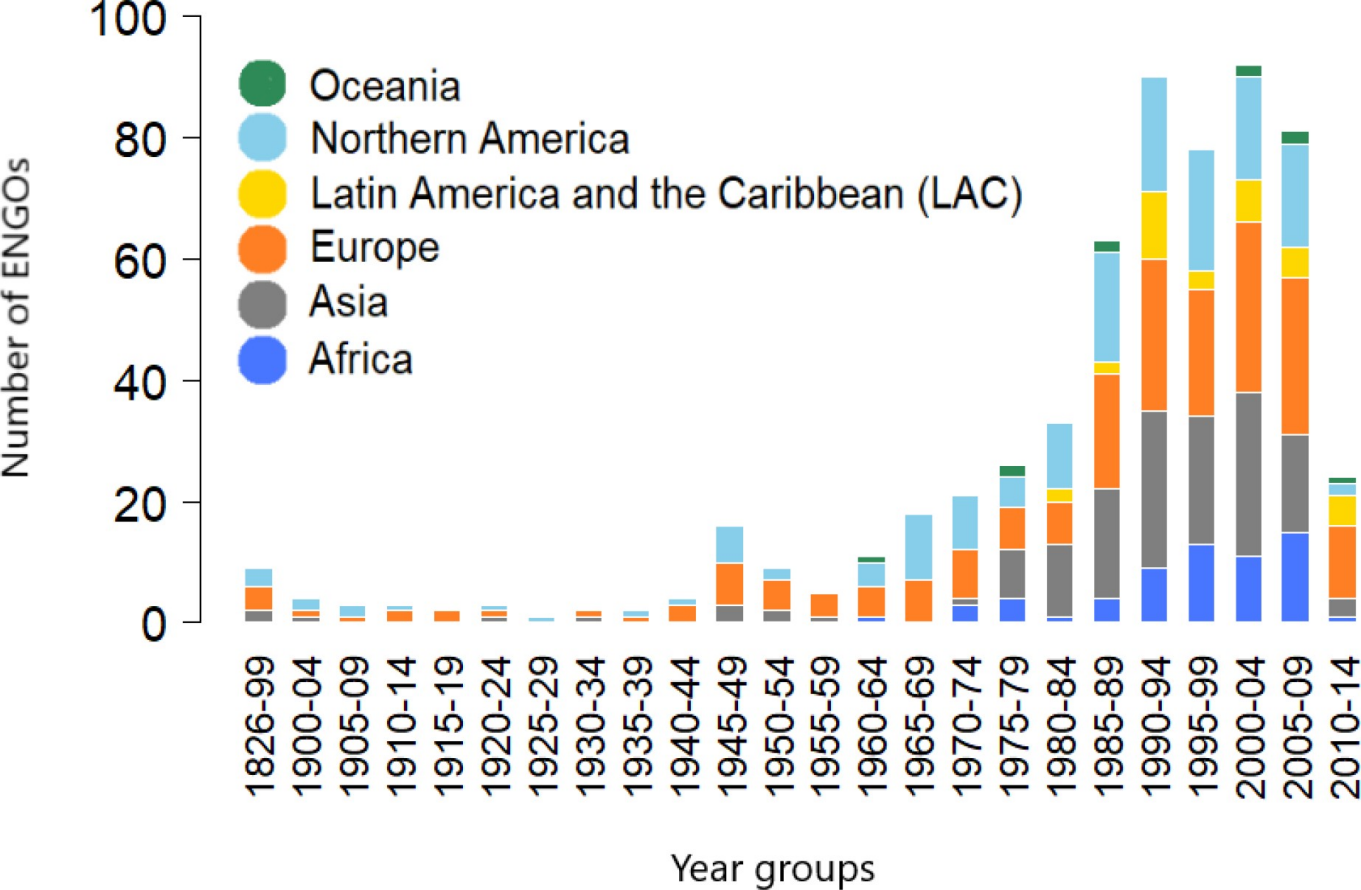
History of ENGO establishment by region (*N* = 601). Data are aggregated into five-year intervals beginning in 1900 (second bar). Groups established in 1826–1899 are shown in the first bar.

The oldest ENGOs were established in the 19th century, predominantly in Europe, Northern America, and Asia (Fig 1). Few ENGOs were established in the early 20th century. An uptick accompanied the expansion of international organization following World War II. Many ENGOs were subsequently established with the emergence of the environmental movement in Europe and Northern America in the 1960s-1970s. A recent spike in ENGO formation, beginning in the late 1980s, coincides with the 1992 Rio Conventions (Earth Summit) and includes increased rates of ENGO establishment in the Global South (S5 Table in S1 Appendix). While roughly half the ENGOs in Northern America (51%) and Oceania (50%) were established before 1990, ENGOs in Africa (79%) and LAC (89%) were established predominantly between 1990–2014.

ENGOs in our dataset are unevenly distributed geographically. Over 80% of the ENGOs in our sample are based in Asia (23.3%), Europe (33.6%) or Northern America (24.6%). Relatively few ENGOs are based in Africa (11.2%) and LAC (5.4%), although ENGO establishment in these regions increased over the past three decades. Oceania hosts the fewest ENGOs (1.9%) (Fig 1).

### Structural power: Financial capacity and human resources

We obtained the number of employees for two-thirds of ENGOs in our sample (*N* = 432). The largest ENGOs by workforce are based primarily in Europe and Northern America (Table 2). Europe houses both the largest ENGO (GIZ, Germany) and some of the smallest ENGOs, which work exclusively through volunteers (e.g., Architecture Sans Frontières, Sweden). (GIZ is a state-owned development agency, but participates in the ENGO constituency at UNFCCC; see S1 Appendix, Methods for further discussion). The median number of employees ranges regionally from 7 in Oceania to 27 in LAC.

**Table 2:** ENGO human resources and financial capacity by region. ENGOs for which data are unavailable are recorded as NA.

Region	*N*	(A) Number of employees				(B) Budget in 2016 USD (thousands)			
		*Min*.	*Median*	*Max*.	*NA*	*Min*.	*Median*	*Max*.	*NA*
Africa	76	1	11	99	34	7	385	274,684	47
Asia	158	1	18	7,101	83	14	1,024	1,105,110	98
Oceania	13	2	7	150	6	398	2,145	13,505	10
Europe	228	0	19	17,319	72	14	3,623	2,180,556	101
Northern America	167	0	20	3,824	36	264	7,788	1,018,000	66
Latin America and the Caribbean	37	0	27	103	16	11	3,322	170,420	21
All	679	0	17	17,319	247	7	3,106	2,180,556	343

We located budget data for half the ENGOs in our dataset (*N* = 336). Northern American ENGOs have the highest median budgets, followed by Europe (Table 2). Overall, distribution of financial resources is highly skewed: 6% of ENGOs (*N* = 21) hold 75% of the total sectoral budget, and the wealthiest 20% (*N* = 67) hold 94% of financial resources.

We created a structural power index as a combined measure of human resources and financial capacity. Relative to the full sample, the structural power index (*N* = 276) over-represents Northern American ENGOs (+8.4%) and under-represents Asian ENGOs (-9.2%) (S2 Table in S1 Appendix). African ENGOs comprise 9.1% of the index, but 26% of the low-power quartile, with 72% of African ENGOs having low structural power (Table 3). Asian ENGOs have greater human and financial resources than African groups, and are concentrated in the medium-low quartile. Structural power is disproportionately concentrated in Northern America and Europe, which account for 81.2% of high and medium-high power groups. Northern American ENGOs comprise 43.5% of the high-power quartile, and 62.6% of Northern American groups are in the top half of the index. European ENGOs are heterogeneous, with balanced distribution across quartiles from low- to high-power groups. One-third of LAC ENGOs are in the top half of the index, but larger and better-funded LAC organizations are likely over-represented in our sample.

**Table 3:** Structural power index by region (*N =* 276). Cells are shaded according to rank within the quartile. Darker cells indicate regions with greater representation in the quartile.

Region	*N*	Number of ENGOs per quartile			
		Low	Medium-Low	Medium-High	High
Africa	25	18	3	3	1
Asia	39	9	14	9	7
Oceania	3	1	0	2	0
Northern America	91	10	24	27	30
Europe	106	27	24	27	28
Latin America and the Caribbean (LAC)	12	4	4	1	3
**Total**	**276**	69	69	69	69

### Environmental discourse

**Fig 2:**
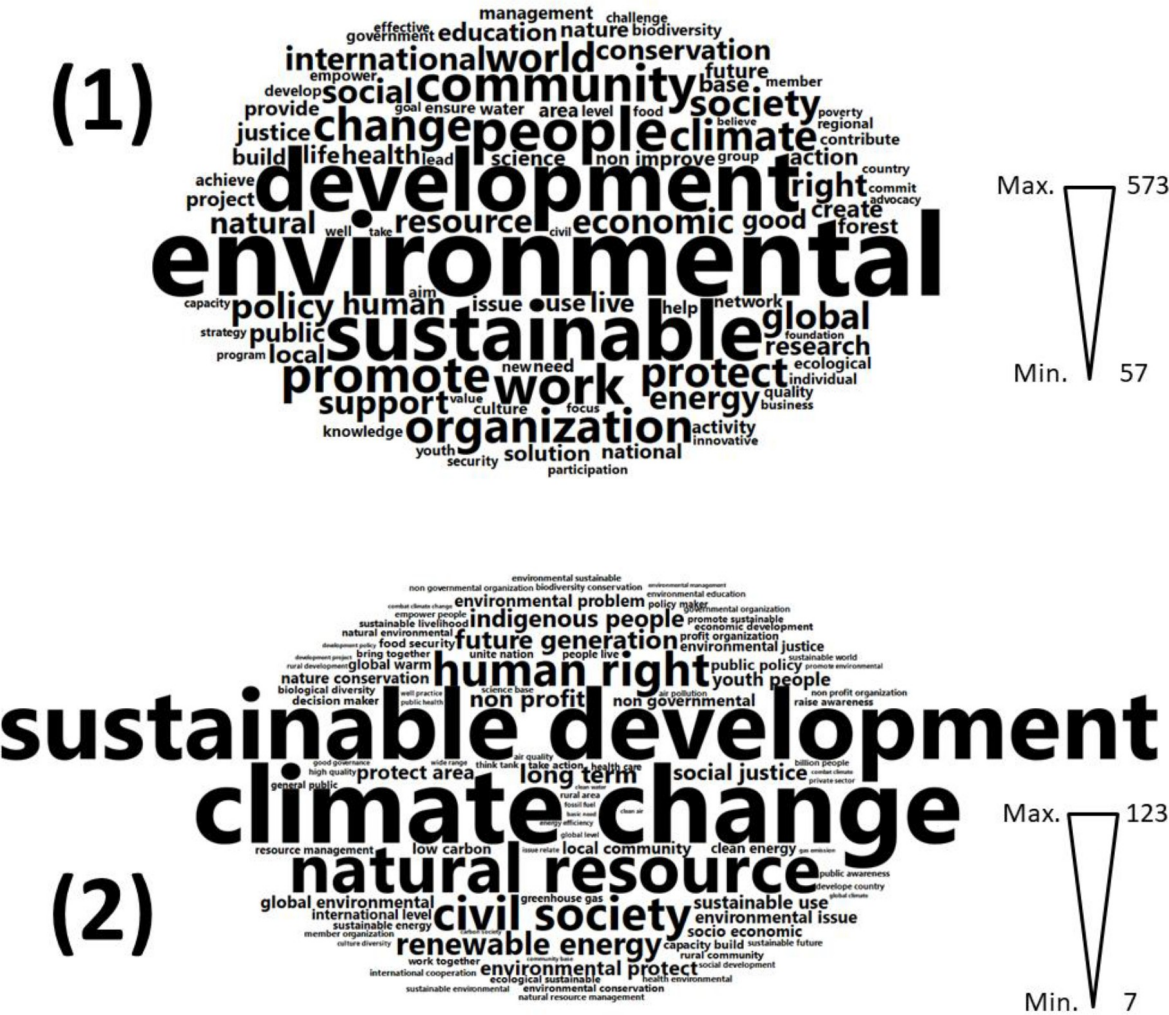
**Word clouds showing relative frequency of (1) single words, and (2) word combinations in all mission statements**. Word permutations were lemmatized to a single root and are displayed under the most frequently occurring variant. Words are sized proportionally to frequency of occurrence. Word clouds are scaled internally; therefore, word sizes in (1) and (2) are not directly comparable.

First-stage content analysis of ENGO mission statements examined word frequency (Fig 2). The most frequently occurring words across ENGO mission statements are *environmental* (573 hits), *development* (432), and *sustainable* (409), and the most frequently occurring word combination is *climate change* (123). Use of *environmental* exceeds occurrence of related terms like *nature* (110) or *ecological* (80). Through inductive coding, we classified words and word combinations into 11 categories: **actions**, **activities**, **capabilities**, **economic** terms, **environmental** terms, geographical **levels**, **places**, **political** terms, **social** terms, **time**, and **values** (S1 Appendix).

Frequency counts do not account for context, which may alter meaning; nonetheless, frequently-occurring terms indicate key concerns in environmental discourse. The most frequent **environmental** terms, beyond *environmental* (573) itself, include *sustainable* (409), *climate* (216), *resource* (171), *conservation* (143) and *nature* (110). *Forest* appears 106 times, and *water* 85 times. Key **political** terms include *policy* (181), *international* (163), *right* (162), *public* (131), and *justice* (118). Prominent **social** terms are *people* (304), *community* (260), and *society* (193). *Culture* is mentioned 79 times. Frequently-occurring **economic** terms include *work* (273), *economic* (175), *resource* (171) and *energy* (169). *Business* appears 63 times, and *sustainable use* (21) is a common word combination. The prominent words *development* (432) and *sustainable* (409) belong to multiple categories.

Priority **actions** include to *promote* (250), *protect* (216), *support* (172), *create* (122), *build* (113) and *provide* (105). Priority **activities** focus on *research* (131), *science* (106), *networks* (89), *management* (93), and *advocacy* (60). *Projects* (104) are mentioned more frequently than *programs* (58), and *nature conservation* (18) is the most common word combination, followed by *capacity building* (14). **Capabilities**, defined as basic necessities or key components of human flourishing [69], emphasize *health* (132), *education* (128), *knowledge* (76), *security* (67), and *food* (66). *Human rights* are mentioned 54 times, and *food security* 14 times. Prominent **values** include *quality* (74) and *effectiveness* (69). *Value* appears 63 times, and *environmental justice* (16) is the most common word combination, with *air quality* (9) or *clean air* (7) also a key concern.

Geographical **levels** most frequently mentioned are *world* (204), *global* (183), and *international* (163). The *local* (126) level appears next, followed by *national* (104) or *country* (62), *individual* (71), and *regional* (70). Some mission statements refer to specific geographical **places**, such as *Hong Kong* (6), *Latin America* (6), and the *United States* (6). Language referring to **time** is focused on the *future* (107), and *youth* are mentioned 70 times. *Future generations* (31) are frequently invoked, as is a *sustainable future* (10).

Second-stage content analysis comprised principal component analysis (PCA) where extremes of the two primary principal components (PCs) were used to identify four environmental discourses (Table 4). Based on their strongest-loading indicator terms, we labeled the four discourses: (1) Environmental Management, (2) Climate Politics, (3) Environmental Justice, and (4) Ecological Modernization. These labels are influenced by correspondence of indicator terms to discourses identified in existing literature, making our discourse labeling a process of ‘inference to the best explanation.’ Table 5 summarizes existing discourse typologies. Table 4 provides indicator terms and a narrative description of the core of each discourse in our typology.

**Table 4:** Typology of environmental discourses. Discourses are identified inductively through PCA and labeled according to 15 indicator terms that drive variation on the PC axes.

Discourse	PC	Indicator Terms	Narrative Description
(1) Environmental Management	PC1+	environmental management; air quality; environmental conservation; nature conservation; protect area; conservation; promote environmental; sustainable environmental; capacity build; nature resource management; forest; environmental sustainable; science; international cooperation; sustainable energy	Sustainably managing and conserving the environment and natural resources through science, capacity building, and international cooperation, with a focus on protected areas, air quality, forests, and energy
(2) Climate Politics	PC1-	level; change; local; international; work; good; national; government; climate change; civil; future; global; social; climate; take	Working with government and civil society across multiple levels from local to international to address global climate change and build a good future
(3) Environmental Justice	PC2+	right; live; people; nature resource; culture diversity; human right; indigenous people; nature; culture; human; justice; empower; resource; people live; community	Pursuing justice by empowering people and communities to live in a way that respects nature and cultural diversity and guarantees human rights and the rights of indigenous peoples
(4) Ecological Modernization	PC2-	energy; take; business; climate change; climate; greenhouse gas; policy; renewable energy; issue; greenhouse gas emissions; civil society; global climate; national; civil; innovative	Working with business and civil society to develop policy and pursue innovative responses to global climate change by focusing on greenhouse gas emissions and renewable energy development

**Table 5:** Environmental discourse typologies. Existing typologies are compared to our typology of four discourses. Discourses identified by other studies are placed in the column of the discourse(s) from this study to which they bear the strongest affinity. Discourses without clear affinity to our typology are categorized as ‘Other.’ See S1 Appendix, Definitions for discourse definitions from selected studies.

*Authors*	*Methods*	*Discourses*				
		Environmental Management	Climate Politics	Ecological Modernization	Environmental Justice	*Other*
Herndl and Brown 1996 [38]	deductive, semiotic triangle	Regulatory				Poetic
						Scientific
Dryzek 1997 [31]	inductive-deductive, “twenty years of working and teaching”	Survivalism		Sustainability	Green Radicalism	
			Environmental Problem Solving			
Brulle 1996 [17]	inductive, literature review	Conservation			Political Ecology	Ecocentrism
						Deep Ecology
		Preservation			Ecofeminism	
Martinez-Alier 2002 [35]	inductive, methods not specified	Cult of the Wilderness		Gospel of Eco-Efficiency	Environmen-talism of the Poor	
Bäckstrand and Lövbrand 2006 [36]	inductive, methods not specified	Green Governmentality	Civic Environ-mental-ism (reform-ist)	Ecological Modernization	Civic Environmen-talism (radical)	

PCA indicates that Environmental Management is the most commonly engaged discourse by ENGOs (loading positively on the first PC), but it does not, on its own, meaningfully explain variance among ENGOs. Beyond a generalized Environmental Management discourse, discursive variance is best explained by the following three discourses: Climate Politics, Environmental Justice, and Ecological Modernization.

**Fig 3:**
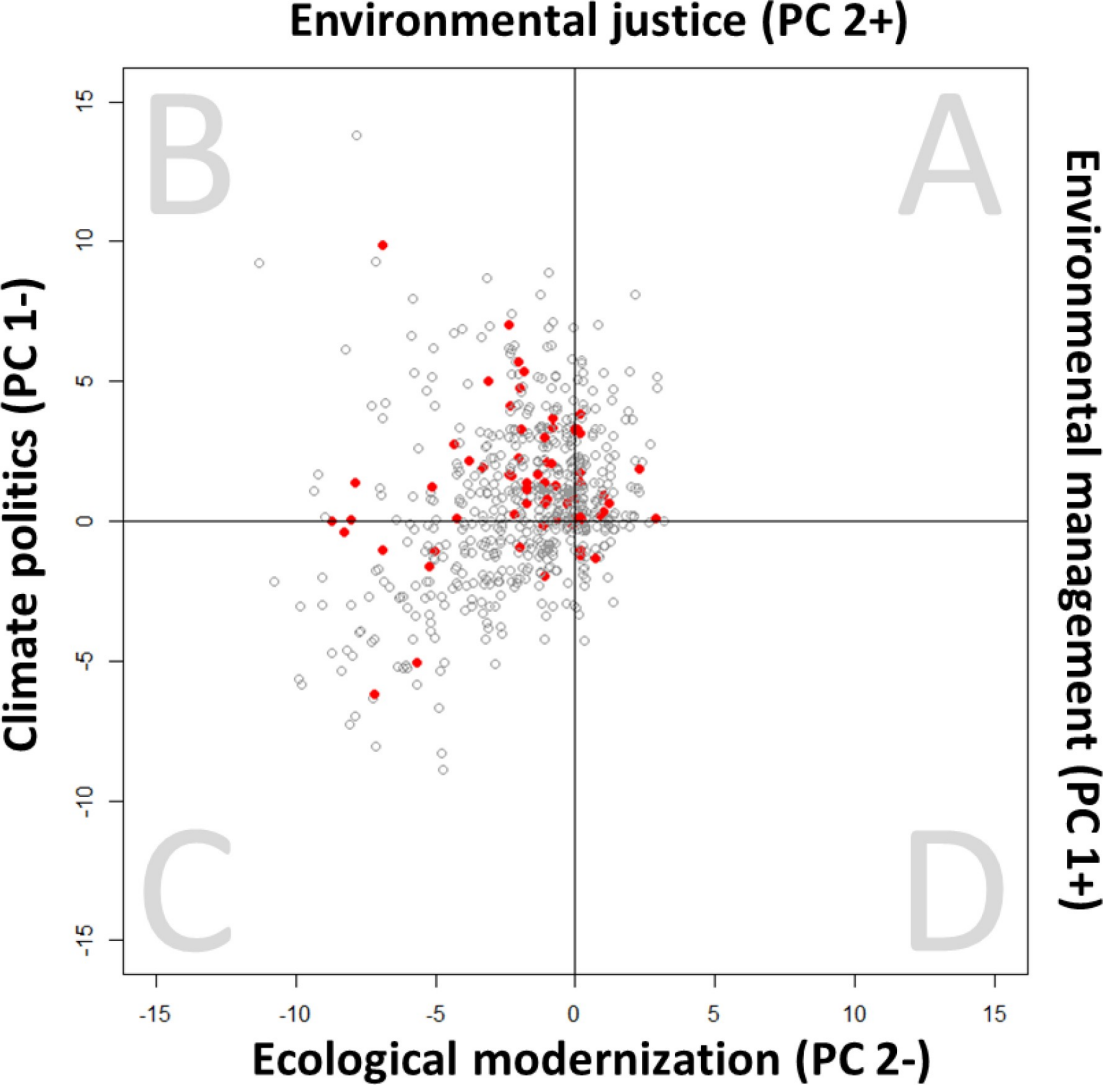
Topology of the ENGO sector. Each circle represents one ENGO (*N* = 679) and its position within the discursive field. Red circles indicate ENGOs in the fourth quartile of the structural power index (high structural power) (*N* = 69).

We mapped a topology of the ENGO sector by plotting the position of individual ENGOs in the discursive field described by our typology (Fig 3; Methods, in S1 Appendix,). Many ENGOs use terminology from all four discourses, and few participate strongly in one discourse to the exclusion of others. Exactly 86% of ENGOs have X and Y absolute values ≤5, resulting in the dense center cluster in Fig 3 (S6 Table in S1 Appendix). Nonetheless, the topology demonstrates clear variation. Climate Politics is a strong concern for many ENGOs: one-fifth of ENGOs (*N* = 143) score ≤-4 on PC1, strongly engaging Climate Politics, while no ENGO scores >3 on PC1 (i.e., engaging Environmental Management without Climate Politics) (S7 Table; S3 Fig in S1 Appendix).

Lack of skew towards Environmental Management does not indicate lack of participation in the discourse. Rather, engagement with Environmental Management discourse is widespread but rarely exclusive, whereas the other three discourses are less widespread and distinguished by ENGOs that engage in these discourses more exclusively, driving variance across the sample. Nonetheless, lack of strong exclusive participation in Environmental Management discourse contradicts superficial characterizations of ENGOs as narrowly concerned with nature conservation and resource management. There is greater balance between ENGOs gravitating towards Environmental Justice versus Ecological Modernization, but some skew towards Environmental Justice, with over 21% (*N* = 148) of ENGOs scoring ≥3 on PC2 (PC2+ Environmental Justice) and ~10% (*N* = 70) scoring ≤-3 (PC2- Ecological Modernization). An ENGO engaging with Environmental Justice discourse is less likely to engage with Ecological Modernization, and vice versa, since these discourses form opposite poles of PC2.

High-profile ENGOs combining Climate Politics and Environmental Justice (quadrant B), include Friends of the Earth International (-8,+14; Netherlands), Oxfam International (-3,+5; UK), and CARE International (-2,+4; Denmark). Those tending to Environmental Management and Environmental Justice (quadrant A) include Wildlife Conservation Society (+2,+2; USA) and Fauna & Flora International (+1,+1; UK). High-profile ENGOs combining Climate Politics and Ecological Modernization (quadrant C) include GIZ (-7,-1; Germany) and the United Nations Foundation (-6,-5; USA). Few ENGOs strongly combine Ecological Modernization with Environmental Management. Quadrant D groups include Greener Impact International (1,-1; Ghana), and Kalahari Conservation Society (1,-1; Botswana).

Most high-power ENGOs are centrally positioned in the discursive field (28.4% have X and Y coordinates of absolute value ≤1), reflecting engagement with multiple discourses. A plurality of high-power groups is located in quadrant B (43.5%) (S3 Table in S1 Appendix), however, combining Climate Politics and Environmental Justice concerns. Regional topologies (S4 Fig in S1 Appendix) show similar distributions to the global plot (Fig 3). LAC ENGOs may skew towards quadrant A relative to other regions, but the small sample cannot support a strong claim of regional anomaly.

## Discussion

Our results offer confirmation for some common assertions about ENGOs and environmental discourse as well as new insights. Firstly, NGOs participating in global environmental politics are more diverse than conventionally recognized. Beyond ‘green-chip’ conservation organizations considered in most ENGO studies, the ENGO sector includes research groups (e.g., National Institute of Advanced Studies (India)), professional and religious associations (e.g., The Foresters’ Association of Turkey (Turkey), Dharma Drum Mountain Buddhist Association (USA)), and human rights and development organizations (e.g., Women’s International League for Peace & Freedom (Switzerland), SNV Netherlands Development Organisation (Netherlands)), which participate in the Rio Conventions and help produce the global environmental agenda. These ‘unconventional’ ENGOs include some of the largest organizations in the sector in terms of human and financial resources, such as Oxfam International (UK) and GIZ (Germany). These groups are included in the dataset thanks to our research design, which replaces arbitrary *a priori* definitions of ENGOs with the population of organizations that participate as ENGOs *in practice* at the Rio Conventions.

ENGOs in our dataset were initially founded primarily in Europe and Northern America beginning in the 19^th^ century. Some of the oldest ENGOs are also located in Asia, however, such as the Manila Observatory (established 1865) and the Bombay Natural History Society (established 1883), where they emerged out of European colonial relations. Modern environmentalism has from the beginning mixed Northern concerns about wilderness preservation with scientific and managerial priorities of colonial government [70]. The three-decade boom in ENGO establishment since the late 1980s coincides with heightened recognition of transboundary and global environmental problems and the neoliberalization of global governance. Our analysis of historical trends in ENGO establishment refers only to surviving ENGOs that enter our dataset and cannot account for ENGO attrition, but these findings are consistent with existing accounts of the history of ENGO establishment (e.g., [12]).

We find a large range of human and financial capacity in our ENGO sample, with a global median of 17 employees and $3.1 million annual budget. The smallest organizations from every region had ≤2 employees, and outside Northern America and Oceania, budget minima were <$15,000/year. While costs of participation in a Rio Convention meeting might be expected to exclude smaller ENGOs, representation of smaller organizations in the population is likely facilitated by donor support. Median number of employees ranges regionally from 7 in Oceania to 27 in LAC. The median LAC organization has a budget-to-employee ratio of $123,037:1, however, while the median Oceanian organization has a ratio of $306,429:1. At the extremes, the median Northern American ENGO has a budget-to-employee ratio of $389,400:1, while the median African ENGO has a ratio of $35,000:1, differing by an order of magnitude. These differences likely reflect both different labor costs and financial flows, such that in general Southern ENGOs employ more people with less money while Northern groups handle more money with fewer employees. This disparity is also indicative of a global division of labor where Northern ENGOs act as donors or coordinators for large projects, while Southern ENGOs are subcontracted for implementation, which requires more labor (cf. [27]). A related point is that our regional analysis of human and financial resources is based on the locations of ENGO headquarters, but it does not reveal where resources are invested. Many Northern ENGOs have employees located internationally and spend substantially on projects in other regions. Further data would be required to reveal the amount of ENGO resources invested in a particular region and the financial relations among ENGOs in our sample.

The content analysis generates numerous insights. The first-stage analysis confirms the dominance of a ‘sustainable development’ frame in global environmental discourse, with climate change as the premier environmental issue. It also indicates priorities on forests, water, and air quality. The global and local levels are privileged over the national or regional, and many ENGOs invoke youth and concern for the future. Business and human rights both figure prominently, though the second-stage analysis demonstrates that these terms are primarily invoked by opposing discourses: Ecological Modernization and Environmental Justice, respectively.

In the second-stage content analysis, our inductive discourse typology identifies an Environmental Management discourse, which is closest to what is traditionally imagined as the ‘environmentalist’ or ‘conservationist’ discourse. While this Environmental Management discourse is broadly shared across the ENGO sector, few ENGOs employ it to the exclusion of Climate Politics. The prominence of Climate Politics is not surprising, given the large cohort of UNFCCC observers. The substantial number of ENGOs specializing in Climate Politics to the exclusion of Environmental Management discourse may partly reflect engagement with climate politics by social or development organizations that have not historically worked on environmental conservation (cf. [71]).

Climate Politics and Ecological Modernization discourses are similar, emphasizing climate change, civil society, and government policy, but instead of combining these discourses, many ENGOs mix Environmental Justice with Climate Politics, likely indicating how human development organizations such as CARE (Denmark) and Oxfam (UK) have taken up Climate Politics. It is significant that these organizations are included in our analysis, since the ENGO sector is often arbitrarily limited to mainline Environmental Management organizations, thus missing the more complex topography of the sector. Those green-chip ENGOs, meanwhile, might stereotypically be expected to combine Environmental Management with Ecological Modernization techno-politics, but instead they are more prominently mixing Environmental Management and Climate Politics with Environmental Justice. Several green-chip ENGOs skew toward Environmental Justice while scoring neutrally on PC1, including Conservation International (0,+4; USA), Sierra Club (0,+3; USA), and The Nature Conservancy (0,+3; USA). This sensitivity to Environmental Justice reflects a shift at mainline organizations after critiques of ‘fortress conservation’ during the early 2000s [7,72], though some argue that practices have not changed in tandem with discourse [22]. With longitudinal mission statement data, future studies could trace dynamics of discursive change (e.g., cascades or tipping points).

ENGO distribution across the discursive field is largely independent of structural power, though high-power organizations skew somewhat towards quadrant B and away from quadrant C relative to the full sample (S3 Table in S1 Appendix), reflecting financial resources targeting climate change and the high structural power of human development organizations engaging in climate politics. While our data allow us to identify heterogeneity and patterns in ENGO discourse, the dense central clustering in Fig 3 underlines that most ENGO mission statements participate in multiple discourses.

### Environmental discourse typology

A major contribution of this study is an empirical typology of environmental discourse derived from a survey of globally-networked ENGOs. Our typology of Environmental Management, Climate Politics, Environmental Justice, and Ecological Modernization is not exhaustive, and other discourses exist. Our typology was developed through inference to the best explanation based on correspondence of discourse indicator terms to existing discursive categories; as such, our typology has similarities with some existing typologies, but also clear differences in the primary types of environmental discourse identified. Table 5 maps a selection of existing environmental discourse typologies onto our typology.

Reviewed typologies agree with our typology on the prominence of Environmental Management and Ecological Modernization perspectives in environmental discourse. Most typologies also attend to Environmental Justice discourse, though often within a vague category of ‘radical’ perspectives [31,36] or through a multiplicity of minor discourses [17]. Martinez-Alier [35] is most explicit in identifying an Environmental Justice discourse (‘environmentalism of the poor’). He observes that scholars of environmentalism have often overlooked this discourse because Environmental Justice-oriented organizations have not always advocated within an environmental idiom [35] (p. 14), which resonates with our argument for studying ENGOs beyond green-chip organizations, while also reinforcing studies describing recent consolidation of a ‘justice frame’ in mainstream environmental politics [73,74]. Existing typologies largely miss Climate Politics discourse, or subsume it within other discourse categories. The plethora of issue linkages to climate change [71] and significance of global climate change for environmental politics in the Anthropocene, however, give substantive support to our finding that Climate Politics should be recognized as an environmental discourse in its own right. Lastly, we do not find prominent poetic or spiritual discourses within global environmental discourse, nor are ecocentrist or deep ecology perspectives prevalent enough to register in our typology.

The prominence of Climate Politics and the ‘sustainable development’ concept, coupled with participation in the Rio Conventions by organizations with diverse social and development agendas, suggests that the ‘Environmental NGO’ sector may now be better understood as a ‘Sustainability NGO’ sector addressing concerns much broader than classic Environmental Management issues.

### Methodological considerations and directions forward

This study presents novel methodological approaches for defining the ENGO population and conducting quantitative content analysis of environmental discourse. We believe this data-driven, inductive strategy for studying the ENGO sector can fruitfully be expanded to complement and improve on our initial analysis. Quantitative content and discourse analysis is an area of active experimentation in environmental and sustainability research fields [39,66–68,75], and careful consideration of the underlying data and mechanics of the quantitative methodology are necessary for accurate interpretation of results. Further methodological refinements will enhance our ability to interpret and communicate results of these methodologies.

Extensions to our dataset could expand coverage of the ENGO universe by including ENGOs participating in other global and regional governance processes, such as the World Conservation Congress or the Convention on International Trade in Endangered Species of Wild Fauna and Flora (CITES), and by developing selection criteria for ENGOs not participating in global networks. Including mission statements in languages other than English would expand coverage and representativeness of the ENGO universe. In this study, we requested English-language mission statements from ENGOs that provided non-English mission statements on their websites. For the globally-networked ENGOs in our population, our procedures produced a sample with geographical and linguistic biases that are detectable but not extreme. Working across multiple languages would correct linguistic bias in the sample and potentially clarify regional differences not detected by our analysis. Multilingual data would introduce numerous complications, however, since quantitative content analysis with words as raw data would require data integration through standardized translation across languages. A research team with native levels of proficiency in each language could conduct a multilingual discourse analysis, although the translation process itself would introduce new biases [76]. Despite logistical challenges, such a study could provide both improved coverage and insights into cross-cultural discursive translation.

Our findings provide a snapshot of the ENGO sector and environmental discourse in the mid-2010s. Expanding the time series of this dataset would enable longitudinal analysis of the development of the ENGO sector and discourse. Longitudinal data on changing participation in global meetings is readily available, but data on change in ENGO mission statements over time would be challenging to assemble for a large sample, though analyses of small samples demonstrate the value of these data [72]. Collection of additional data on financial flows or network connections among ENGOs and between ENGOs and funders [29,30] would enable more fine-grained analysis of power relations and sectoral structure. From a qualitative perspective, future research might develop case studies to apply our discourse typology and compare ENGOs across different discursive quadrants and structural power gradients. Qualitative research may be especially helpful in examining how ENGO actions and tactics relate to mission statements and organizational discourse.

The rapid proliferation of ENGOs over the past three decades within polycentric environmental governance institutions mandates new conceptualizations of environmental politics in a time of radical and uncertain planetary transformation. Empirical analyses of ENGOs and environmental discourse are essential building blocks for substantive theorization of the role of NGOs in global environmental governance and public policy.

## Supporting information

S1 Appendix (DOCX)

S1 Data (XLSX)

S2 Data (CSV)

S3 Data (CSV)
