# Supplementary material for: Environmental non-governmental organizations and global environmental discourse
Source: PLoS One. 2020 May 27;15(5):e0232945. doi: 10.1371/journal.pone.0232945 (PMC7252593; doi:10.1371/journal.pone.0232945)
Supplement: S1 Appendix — (DOCX) [file pone.0232945.s001.docx]

**Supplementary Methods**

***Defining the ENGO population***

We defined our target population of ENGOs as those accredited to the UNFCCC, UNCCD, and CBD. The three Rio Conventions structure civil society participation differently. The UNFCCC Secretariat lists civil society organizations (CSOs) admitted as observers to the UNFCCC process. Admitted groups may register for meetings and self-select into constituencies that mirror ‘major groups’ established in Agenda 21. ENGOs comprise one of these major groups. We downloaded the list of 1,845 UNFCCC-admitted CSOs in May 2016 and included the 745 organizations in the UNFCCC ENGO constituency in our population. The UNCCD Secretariat maintains a similar list of accredited CSOs, but does not distinguish these CSOs by major groups. We obtained the list of the 314 CSOs accredited to UNCCD as of November 2015 and manually classified CSOs accredited to UNCCD as ‘ENGO’ or ‘other’ based on the CSO’s name and official website, where ENGOs are defined as non-governmental organizations engaged in projects or advocacy related to environmental concerns. The CBD does not maintain a general list of accredited CSOs; rather, organizations must apply for observer status for specific CBD meetings. CBD observers are categorized according to ‘major group’ designations. We obtained the list of 273 observer organizations that participated in the 12^th^ CBD Conference of the Parties (COP) in South Korea in October 2014 and included the 108 organizations in the NGO category in our population.

**Example 1:** GIZ is the German state-owned development agency that primarily implements German Government cooperation projects. The organization participates in the ENGO constituency at UNFCCC meetings, and so it is included as an ENGO in our dataset.

(NB: While GIZ is not conventionally considered an ENGO, we choose not to circumvent our methodology by ad hoc excluding organizations participating in the ENGO constituency. ENGOs have diverse relationships with state agencies, and the distinction between the state and civil society is “a line drawn internally within the network of institutional mechanisms through which a social and political order is maintained” [1] (p. 78). How GIZ accreditation and participation alters dynamics in an NGO space would constitute an intriguing empirical question.)

**Example 2:** “ICLEI - Local Governments for Sustainability” has observer status with UNCCD and CBD, as well as with UNFCCC. Under the UNFCCC constituencies, ICLEI participates in ‘Local Governments and Municipal Authorities (LGMA)’ as opposed to ENGOs. We therefore excluded ICLEI from analysis.

**Example 3**: “CAMP Alatoo Public Foundation” was registered as a CSO with UNCCD. Consultation of the organization’s website (<http://en.camp.kg/who-we-are/about-us.html)> revealed that it is a non-profit, non-governmental organization based in Kyrgyzstan pursuing sustainable development in mountain regions of Central Asia. We classified CAMP Alatoo Public Foundation as an ENGO and included it in our analysis.

Fourteen organizations that could not be classified were eliminated from further analysis; details available on request. Registered universities participate variously at UNFCCC as either ENGOs or “Research and independent non-governmental organizations.” We chose to exclude all registered universities from analysis. The final merged list of ENGOs comprised 978 individual organizations (Figure S1).

**
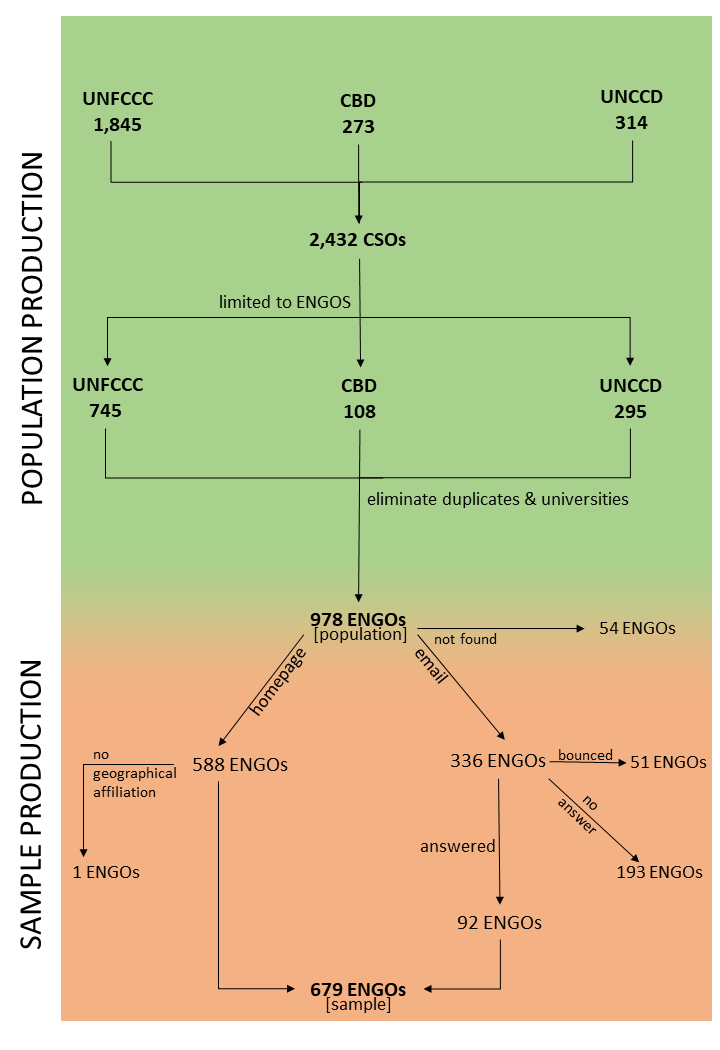
**

**Figure S1**. Flowchart of ENGO population definition and sample selection. Upper half (green) shows the definition of the ENGO population based on the list of civil society organizations with observer status at the three Rio Conventions, producing a consolidated population of 978 ENGOs. Lower half (red) shows the construction of our sample (*N=*679) based on obtaining mission statements via homepages and e-mail.

***Data Collection and Formatting***

We collected attribute data and mission statements for our population of 978 ENGOs from their official websites. Data were collected in 2016, with minor updates to correct errors in October 2018 and November 2019. ENGO homepages were located using URLs provided by the Secretariats, as well as Google searches for the ENGO name. Where we could not find an official website or English-language mission statement, we attempted to contact the organization via email. Where an organization had a ‘vision’ statement in addition to a ‘mission’ statement, both were included in the ‘mission statement’ data for our analysis. The final dataset comprises the sample of 679 ENGOs for which we obtained English-language mission statements, representing 69.4% of the ENGO population.

**Table S1.** Geographical Comparison of ENGO Sample to Population.

| **Region** | ***N* (%) of Population (total *N*=978)** | ***N* (%) of Sample (total *N*=679)** | **+/- % Difference** |
| --- | --- | --- | --- |
| Africa | 171 (17.5%) | (11.2%) | -6.3% |
| Asia | 198 (20.2%) | 158 (23.3%) | +3.1% |
| Oceania | 13 (1.3%) | 13 (1.9%) | +0.6% |
| Europe | 295 (30.2%) | 228 (33.6%) | +3.4% |
| Northern America | 187 (19.1%) | 167 (24.6%) | +5.5% |
| Latin America and the Caribbean | 113 (11.6%) | 37 (5.4%) | -6.2% |
| No affiliation | 1 (0.1%) | 0 (0%) | -0.1% |

We also collected data on ENGO nationality, founding year, employees, and budget. Budget data comprise total annual income (revenue) in the most recent available year, and we converted all budget figures to 2016 USD.

We calculated a structural power index that classifies ENGOs by combining budget and employee data (*N*=276). Budget and employee data were normalized to a mean of 0 with all values within one standard deviation of the mean. These two values were averaged together to generate a single index score representing the structural power of each ENGO. The index values were divided into quartiles for analysis.

***Content Analysis***

We carried out a quantitative content analysis of ENGO mission statements using MaxQDA [2]. Since ENGOs revise their mission statements with some frequency [3], this analysis should be considered a cross-sectional snapshot of environmental discourse in the mid-2010s. The first stage of the analysis assessed the frequency of occurrence of individual words across all mission statements. We applied a stop-list to exclude words that do not represent meaningful contributions to the discourse (e.g., ‘is,’ ‘and,’ ‘the’) and generated a full list of individual words ranked by frequency of occurrence across all statements. We selected approximately the top 10% of most frequently occurring words to generate a final list of 518 words. We then produced a lemmatization list to aggregate different parts of speech with a common root (e.g., ‘conserve,’ ‘conservation,’ ‘conserving’) as well as verb forms appearing in different conjugations (e.g., ‘gave,’ ‘given,’ ‘give’) and singular versus plural forms. Words were lemmatized to the most commonly occurring variant. A lemmatized ‘word’ thus captures multiple permutations of a specific concept. Using our lemmatizations, we generated a new list of the 100 most frequent single words and a list of the 100 most frequent meaningful word combinations of two or three words (e.g., climate change, natural resource management) across all mission statements. Single words and word combinations are counted separately, meaning that the single word count reflects total usage and summing single word (e.g., ‘climate’) and word combination (e.g., ‘climate change’) occurrences would result in double counting. We report results on the total number of occurrences of these words (list A), as opposed to the number of mission statements within which each word occurs (list B). A Kendall ranked-list correlation analysis [4] of the 100 words and 100 word combinations occurring in the largest number of mission statements (list B) with our chosen list A indicates that the single word lists received a Kendall tau correlation rank of 0.805, and the combination word list of 0.7353, both with p-values <0.0001. Thus, the choice of which list method to use has a largely negligible effect on overall outcomes.

The three authors independently open-coded the word and word combination lists to generate inductive categories, which were combined to generate a master code list (below). The three authors independently re-coded word and word combination lists using the master codes. Final classifications of words and word combinations into categories are based on agreement between at least two coders. Agreement between at least two coders across all assigned codes was 71%. The master codes and definitions are as follows:

**environmental** Having to do with the Earth System, geophysical features, or non-human life

**social** Having to do with humans or their interactions with each other

**economic** Having to do with production, exchange, provisioning, or profit

**political** Having to do with the exercise of power, governance, or policy

**action** Effecting a change in a particular way or through a particular strategy, usually a verb

**level** A designation of geographical organization in human or environmental systems

**place** A specific geographical space

**activity** An organized function performed by an organization

**time** Indicating temporality

**value** Indicating positive or negative judgment or a goal to be pursued

**capability** A necessity or key component of human survival or flourishing

In the second stage of analysis, we generated a binary dataset indicating the occurrence (yes or no) of each of the most frequent words and word combinations in all mission statements. We applied principal component analysis (PCA) to the binary dataset of word occurrence to identify clusters of terminology that explain variation in ENGO discourse.

We identified with a scree plot [5–7] two principal components (PCs) of 88 terms each that together explain ~10.6% of the variance in terminology across ENGO mission statements. These PCs are used to generate indicator words for the inductive identification of distinct discourses. Each PC is interpreted as a spectrum: terms loading most strongly positive on a PC are more likely to co-occur in a mission statement with other terms at the positive end of the spectrum, and less likely to co-occur with terms that load strongly negative. Two PCs thus produce four clusters of terminology (two from each PC), which we take as the terminological nuclei differentiating four environmental discourses. We then used the top 15 words in each cluster as indicator words for scoring each mission statement. These clusters are used as indicators for the four discourses as opposed to single words because no single word was able to explain a significant portion of the variation of a PC, but multiple words or clusters of words in combination better explain thematic variation in the content analysis. The indicator terms comprise 34% of each PC, i.e., the 60 strongest-loading terms on the two PC spectrums (176 terms total).

We coded each mission statement for the presence or absence of the 60 words from the four discourse clusters. The two PCs are imagined as X and Y axes, and each mission statement receives a value on each axis. Statements receive +1 for each word they contain from the positive cluster of the PC and -1 for each word they contain from the negative cluster (Table 2). A statement containing all words from the positive cluster and no words from the negative cluster receives a score of +15, indicating strong and exclusive usage of that discourse. A statement containing all words from both discourses receives a 0. This statement participates in both discourses, but is not a distinct representative of either. This classification method reflects the logic of PCA, namely, that discourses are defined in contradistinction to each other and are known through actors that employ one set of concepts and not another. The two PC scores are plotted as (X,Y) values to show the distribution of ENGOs across the four quadrants of the discursive field (Figure 3).

**Supplementary Results**

**
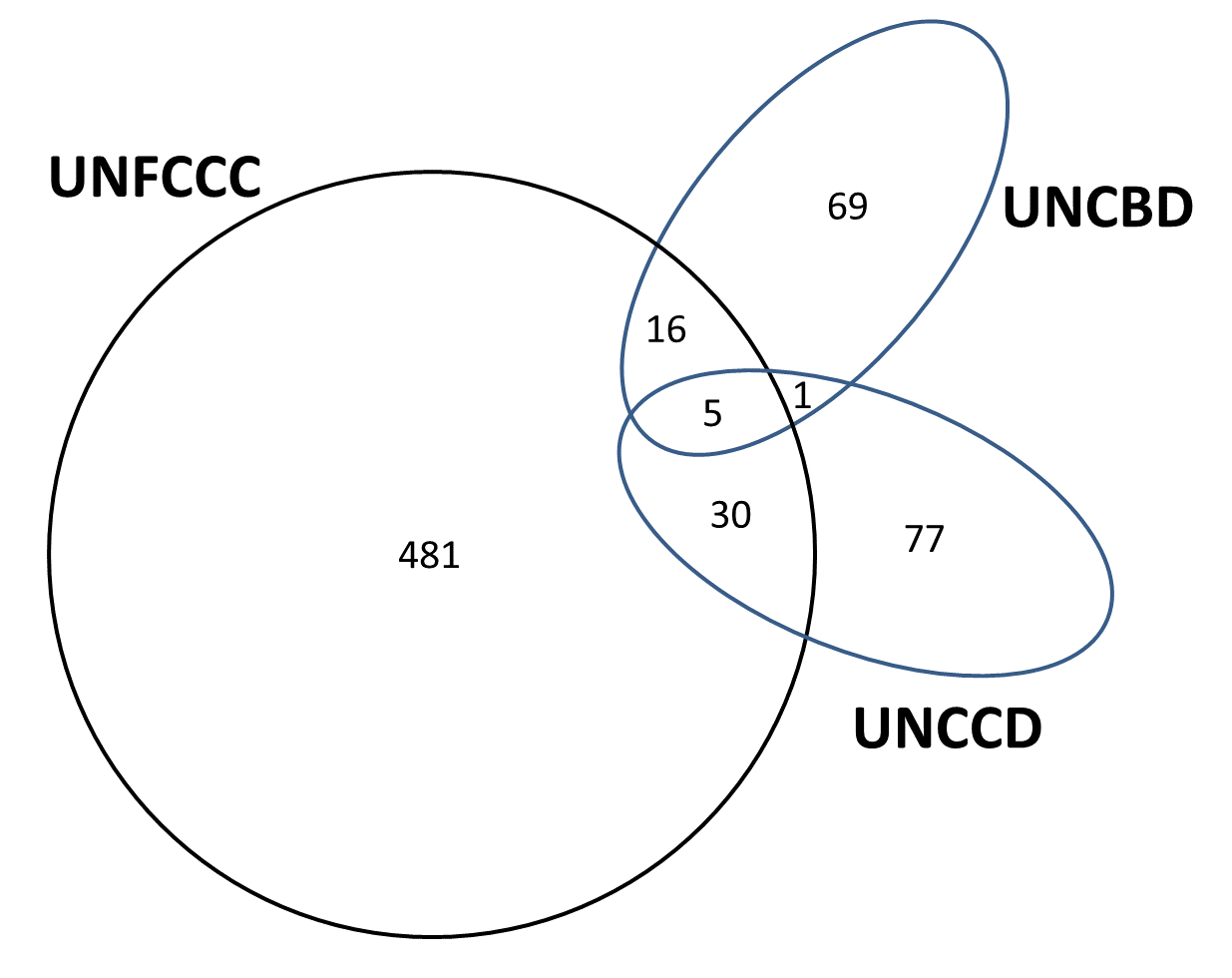
**

**Figure S2**. Observer status of ENGOs in our sample (*N*=679) with the three Rio Conventions: UNFCCC, CBD (COP 12), and UNCCD. Figure produced using euler*APE* [8]. The five ENGOs accredited to all three conventions were Conservation International (USA), Green Asia Network (Republic of Korea), Rainforest Alliance (USA), World Resources Institute (USA), and WWF (Switzerland).

**Table S2.** Geographical skew of Structural Power Index relative to full sample.

| **Region** | **% Full Sample** | **% SPI** | **+/- % Difference** |
| --- | --- | --- | --- |
| Africa | 11.2 | 9.1 | -2.1 |
| Asia | 23.3 | 14.1 | -9.2 |
| Oceania | 1.9 | 1.1 | -0.8 |
| Europe | 33.6 | 38.4 | +4.8 |
| Northern America | 24.6 | 33.0 | +8.4 |
| Latin America and the Caribbean | 5.4 | 4.3 | -1.1 |
| Total | 100 | 100 | 0 |

**
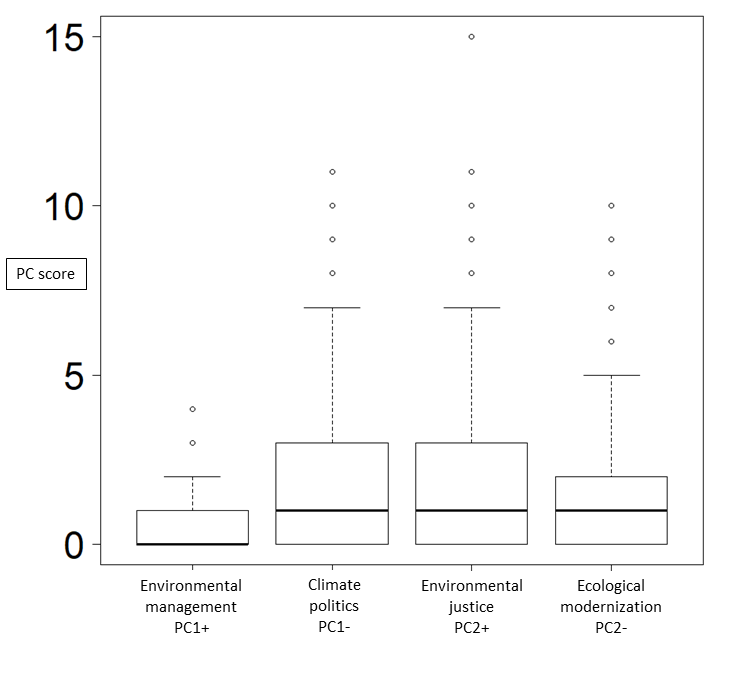
**

**Figure S3**. The distribution of ENGO PC scores for each set of 15 indicator terms. (Raw scores are reported for all ENGOs on each discourse, *N*=679.)

**Table S3.** Distribution of ENGOs in the discursive field (Figure 3).

| **Discursive Orientation** | **Number of ENGOs (%)** | **Number of High-Power ENGOs (%)** |
| --- | --- | --- |
| Environmental Management and Ecological Modernization,  quadrant D | 8 (1.2%) | 1 (1.4%) |
| Climate Politics and Ecological Modernization, quadrant C | 167 (24.6%) | 7 (10.1%) |
| Environmental Management and Environmental Justice,  quadrant A | 48 (7.1%) | 3 (4.3%) |
| Climate Politics and Environmental Justice, quadrant B | 204 (30.0%) | 30 (43.5%) |
| Ecological Modernization,  PC1=0, PC2 negative | 21 (3.1%) | 3 (4.3%) |
| Environmental Justice,  PC1=0, PC2 positive | 89 (13.1%) | 10 (14.5%) |
| Climate Politics,  PC1 negative, PC2=0 | 62 (9.1%) | 7 (10.1%) |
| Environmental Management,  PC1 positive, PC2=0 | 31 (4.6%) | 3 (4.3%) |
| origin (neutral: PC1=0, PC2=0) | 49 (7.2%) | 5 (7.2%) |
| Total | 679 | 69 |

**
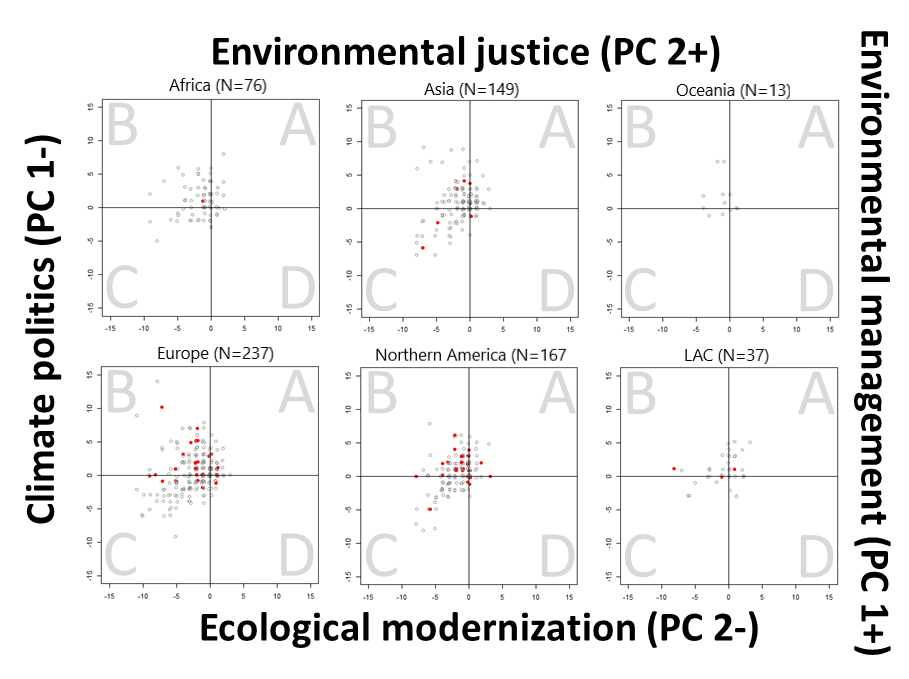
**

**Figure S4.** Regional ENGO topologies with high-power groups denoted by red circles.

**Table S4**. Extreme ENGOs. ENGOs with the most extreme positive and negative values for each PC, making them the strongest examples of each discourse.

| **ENGO** | **(X,Y)** | **Discourse Extreme** | **Mission Statement** |
| --- | --- | --- | --- |
| A SEED Europe (Netherlands) | PC1 -11, PC2 +9 | Climate Politics (PC1-) | Action for Solidarity, Equality, Environment and Diversity, (ASEED Europe) believe that people have the potential to change their lives and communities for the better and we aim to support and empower both groups and individuals who are striving to do so. We believe that the roots of the ecological destruction and poverty which is ravaging our planet today can be traced back to the colonial and imperialist past when the populations and natural resources of entire continents were ruthlessly exploited. We see today’s profit-obsessed ‘free’ market globalisation which is fuelled by the increasing mobility of capital as a continuation of this phenomenon. Our campaigns are based on the need to act in solidarity with the millions of people in the Global South whose already precarious livelihoods are being ruined by unfair competition and disastrous privatisation programmes imposed by bodies like the International Financial Institutions and trade agreements in the name of ‘free’ trade and globalisation. So far, globalisation has led to more inequality and the concentration of resources into fewer hands. ASEED Europe believes that everybody counts and that decisions that can make or break lives should be taken locally, not on the stockmarkets of New York and London or behind the closed doors of international financial institutions. We support the rights of individuals and communities to determine their own lives and so we work with groups which promote local democracy and social justice as well as taking these principles as a starting point in our own campaigns. Our commitment to equality is reflected in our non-hierarchical working practises, whereby decisions are made by consensus. One of ASEED Europe’s most fundamental standpoints is that humans are a part of nature, not apart from it. Modern economic systems, regardless of their political hue, continue to regard nature as a resource to be exploited and recent treaties aimed at slowing down ongoing environmental degradation, climate change and the alarming loss of bio-diversity are doomed to failure because short term economic growth is always given precedence over the long term future of the planet. It is imperative that we develop new, sustainable alternatives to our current consumption patterns. ASEED Europe’s campaigns focus on exploring alternatives and networking with groups working to promote viable alternatives and sustainable development. ASEED Europe strives for the preservation of both cultural and biological diversity. We reject and work towards ending discrimination based on age, class, disability, gender, race, religious beliefs and sexual preference. Vital tools that we use to realise our aims are: the promotion of grass root organisation, education, mobilisation and non-violent direct action so that people can act to achieve social justice and environmental integrity on both the local and global level. |
| Norwegian Forum for Environment and Development (Norway) | PC1 -11, PC2 -2 | Climate Politics (PC1-) | Our main goal is to protect nature and environment so that human activity does not exceed the tolerance limits of our planet. We are concerned with a wide range of issues in environmental and nature conservation, but work specifically with the areas conservation, climate, energy and transport. Although we have a national agenda, many environmental questions have proven to have an international or even global character. Development issues, resource allocations and international cooperation are very much parts of our everyday activities. |
|  |  |  |  |
| Borneo Tropical Rainforest Foundation (Switzerland) | PC1 +3, PC2 0 | Environmental Management (PC1+) | · To establish and safeguard large protected areas in order to foster conservation of the tropical rainforests of Borneo and other threatened regions.  · To provide efficient management, finance and infrastructure for the strict preservation of these protected areas as reservoirs of biological diversity for ecological, genetic, economic, scientific and educational purposes.  · To raise awareness of the planetary importance of rainforest protection and its positive impacts for humanity. |
| Naurzum - Non-governmental Ecological Organisation  (Kazakhstan) | PC1 +3, PC2 +3 | Environmental Management (PC1+) | The fundamental goal of “Naurzum” is to unite the efforts of its members in the name of nature conservation, harmonization between people and nature, assistance and development of protected areas and community ecological education. |
| Centro de Estudios Biologicos, Medio Ambiente y Recursos Naturales  (Mexico) | PC1 +3, PC2 +5 | Environmental Management (PC1+) | 1.- Promotion and encourage of education, culture, art, science and technology 2.- Support to natural resources management, protection of environment, conservation and restore of ecological balance, promotion of sustainable development in the urban and rural communities 3.- Support to development of indigenous communities Our main project is develop of scientific skills in our students to contribute in their scientific literay. Become in an important Research Center with own laboratories for study the use and conservation of natural resources of mayan zone of Quintana roo, mexico. |
| Civic Exchange (China) | PC1 +3, PC2 0 | Environmental Management (PC1+) | Civic Exchange is an independent Hong Kong-based public policy think tank that was established in September 2000 by Ms Christine Loh and Ms Lisa Hopkinson. With the mission to advance civic education and engage society to shape public policy, Civic Exchange undertakes research in air quality, nature conservation and urban environment. Shaping a liveable and sustainable Hong Kong to enhance our sense of wellbeing. |
| Gordon E. and Betty I. Moore Foundation  (USA) | PC1 +3, PC2 0 | Environmental Management (PC1+) | We foster path-breaking scientific discovery, environmental conservation, patient care improvements, and preservation of the special character of the Bay Area. |
| Natural Lands Trust (USA) | PC1 +3, PC2 +5 | Environmental Management (PC1+) | Natural Lands Trust is a non-profit land conservation organization dedicated to protecting the forests, fields, streams, and wetlands that are essential to the sustainability of life in eastern Pennsylvania and southern New Jersey. We apply a comprehensive approach to conservation that includes permanently protecting natural areas, providing leadership in natural resource management, and creating opportunities for people to connect to and learn from nature.Natural Lands Trust is a non-profit land conservation organization dedicated to protecting the forests, fields, streams, and wetlands that are essential to the sustainability of life in eastern Pennsylvania and southern New Jersey. We apply a comprehensive approach to conservation that includes permanently protecting natural areas, providing leadership in natural resource management, and creating opportunities for people to connect to and learn from nature. |
|  |  |  |  |
| Global Climate Forum e.V. (Germany) | PC1 -5, PC2 -9 | Ecological Modernization (PC2-) | Human greenhouse gas emissions change the Earth's climate in ways that the changed climate may dangerously interfere with humankind. Climate change is therefore considered to be one of the biggest challenges facing humanity. No single solution will suffice to deal with it. This challenge has to be jointly tackled by science, policy, business and industry, NGOs, and the general public. Numerous academic studies have been carried out on possible adaptation and mitigation policies. However, most of these studies have not fully considered the needs and perceptions of stakeholders affected by or effecting climate change. Industry has made considerable efforts in reducing emissions, developing renewable energy technologies and contributing to emissions trading schemes. Most of this work, however, has been similarly divorced from academia. No clear picture has emerged of the advantages and disadvantages of the different mitigation and adaptation options, let alone a consensus on a reasonable strategy to pursue. The Global Climate Forum is a platform for joint studies and science-based stakeholder dialogues on climatic change. GCF brings together representatives of different parties concerned with the climate problem. The core activity of the Forum is to define and carry out joint studies; these shall provide arguments for long-term climate mitigation and adaptation policies leading ultimately towards a sustainable development path. |
|  |  |  |  |
| Friends of the Earth International (Netherlands) | PC1 -8, PC2 +14 | Environmental Justice (PC2+) | Our vision is of a peaceful and sustainable world based on societies living in harmony with nature. We envision a society of interdependent people living in dignity, wholeness and fulfilment in which equity and human and peoples' rights are realized. This will be a society built upon peoples' sovereignty and participation. It will be founded on social, economic, gender and environmental justice and be free from all forms of domination and exploitation, such as neoliberalism, corporate globalization, neo-colonialism and militarism. We believe that our children's future will be better because of what we do. To collectively ensure environmental and social justice, human dignity, and respect for human rights and peoples' rights so as to secure sustainable societies. To halt and reverse environmental degradation and depletion of natural resources, nurture the earth's ecological and cultural diversity, and secure sustainable livelihoods. To secure the empowerment of indigenous peoples, local communities, women, groups and individuals, and to ensure public participation in decision making. To bring about transformation towards sustainability and equity between and within societies with creative approaches and solutions. To engage in vibrant campaigns, raise awareness, mobilize people and build alliances with diverse movements, linking grassroots, national and global struggles. To inspire one another and to harness, strengthen and complement each other's capacities, living the change we wish to see and working together in solidarity. |


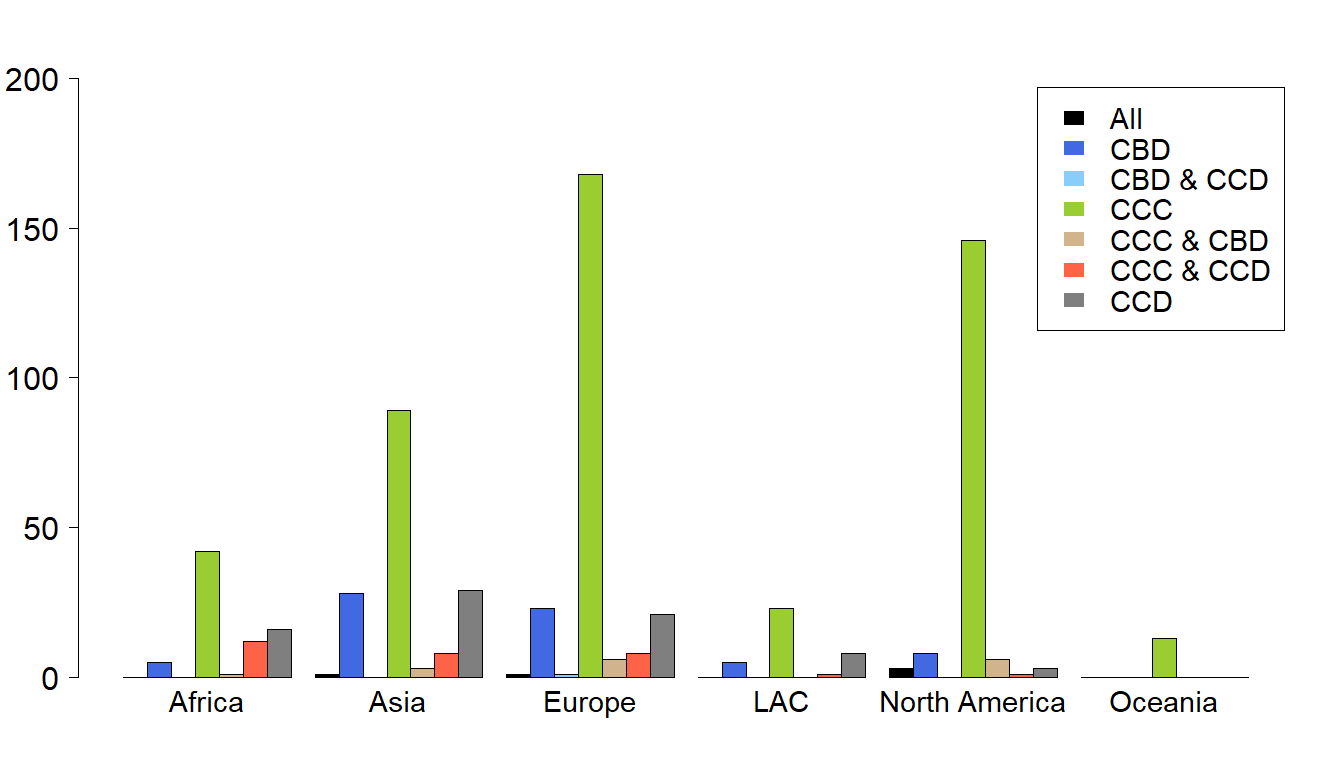


**Figure S5.** Number of ENGOs with observer status with each UN convention by region (*N*=679).

**Table S5.** ENGOs founded before and after 1990 by region.

| **Region** | **Total** | **Founded pre-1990** | **Founded 1990-2014** |
| --- | --- | --- | --- |
| Africa | 62 | 13 | 49 |
| Asia | 143 | 50 | 93 |
| Oceania | 10 | 5 | 5 |
| Europe | 198 | 85 | 113 |
| Northern America | 153 | 78 | 75 |
| Latin America and Caribbean (LAC) | 35 | 4 | 31 |
| Total | 601 | 235 | 366 |

**Table S6.** ENGOs by maximum absolute value of either (X,Y) coordinate.

| **Maximum absolute value of either (X,Y) coordinate** | **Number of ENGOs** | **Percentage of ENGOs** |
| --- | --- | --- |
| **0** | 49 | 7.2 |
| ≤**1** | 207 | 30.5 |
| ≤**2** | 335 | 49.3 |
| ≤**3** | 457 | 67.3 |
| ≤**4** | 528 | 77.8 |
| ≤**5** | 584 | 86.0 |
| ≤**6** | 616 | 90.7 |
| ≤**7** | 644 | 94.8 |
| ≤**8** | 661 | 97.3 |
| ≤**9** | 672 | 99.0 |
| ≤**10** | 676 | 99.6 |
| ≤**11** | 678 | 99.9 |
| ≤**12** | 678 | 99.9 |
| ≤**13** | 678 | 99.9 |
| ≤**14** | 679 | 100 |

**Table S7**. ENGO skew towards Climate Politics (PC1-).

| PC1 score | Number of ENGOs |
| --- | --- |
| ≤0 | 592 |
| ≤-1 | 433 |
| ≤-2 | 302 |
| ≤-3 | 215 |
| ≤-4 | 143 |
| ≤-5 | 102 |
| ≤-6 | 66 |
| ≤-7 | 44 |
| ≤-8 | 25 |
| ≤-9 | 12 |
| ≤-10 | 5 |
| ≤-11 | 2 |

**Definitions: Discourse definitions from existing typologies**

Herndl and Brown (1996) [9]:

1. *Regulatory Discourse*
   1. “This discourse usually regards nature as a resource, one among many others, to be managed for the greater social welfare. In many ways this discourse is the legacy of Gifford Pinchot’s vision of a utilitarian management of natural resources…. As a result, we call this an ethnocentric discourse, one devoted to negotiating the benefits of environmental policy measured against a broad range of social interests” (p. 10-11).
2. *Scientific Discourse*
   1. “Within this discourse, nature is usually regarded as an object of knowledge constructed through careful scientific methodology. Because this discourse locates the human researcher as outside and epistemologically above nature, we call this anthropocentric discourse, one grounded in its faith in the human ability to come to know nature’s secrets” (p. 11).
3. *Poetic Discourse*
   1. “... the language we use to discuss the beauty, the value, the emotional power of nature. In this discourse, nature is usually regarded as a spiritual or transcendent unity. Because this discourse largely considers humanity as a part of nature and seeks to locate human value in a harmonious relation to the natural world, we call this an ecocentric discourse” (p. 12).

Dryzek 1997 [10]:

1. *Environmental Problem Solving*
   1. “... is defined by taking the political economic *status quo* as given but in need of some adjustment to cope with environmental problems, especially via public policy. Such adjustments might take the form of extension of the pragmatic problem-solving capacities of liberal democratic governments by facilitating a variety of environmentalist inputs to them; or of markets, by putting price tags on environmental harms and benefits; or of the administrative state, by institutionalizing environmental concern and expertise in its operating procedures” (p. 13-14)
2. *Survivalism*
   1. “The basic idea is that continued economic and population growth will eventually hit limits set by the Earth’s stock of natural resources and the capacity of its ecosystems to support human agricultural and industrial activity. The limits discourse is radical because it seeks a wholesale redistribution of power within the industrial political economy…. It is prosaic because it can see solutions only in terms of the options set by industrialism, notably, greater control of existing systems by administrators, scientists, and other responsible élites” (p. 14).
3. *Sustainability*
   1. “... is defined by imaginative attempts to dissolve the conflicts between environmental and economic values that energize the discourses of problem solving and limits. The concepts of growth and development are redefined in ways that render obsolete the simple projections of the limits discourse. … More recently, ideas about ecological modernization, seeing economic growth and environmental protection as essentially complementary, have arisen in Europe” (p. 14-15).
4. *Green Radicalism*
   1. “Its adherents reject the basic structure of industrial society and the way the environment is conceptualized therein in favor of a variety of quite different alternative interpretations of humans, their society, and their place in the world. Given its radicalism and imagination, it is not surprising that green radicalism features deep intramural divisions …” (p. 15).

Brulle 1996 [11]: all definitions from Table 1 (p. 64-66)

1. *Conservation*
   1. “Nature is a collection of parts that function like a machine.”
   2. “Humans need to use natural resources to maintain society.”
   3. “Nature can be managed through use of technical knowledge by professionals.”
   4. “The proper management philosophy for nature is to realize the greatest good for the greatest number of people over the longest period of time.”
2. *Preservation*
   1. “Natural systems are self-creating evolutionary wholes that cannot be reduced to the sum of their parts. Hence nature is an intact organism.”
   2. “Human actions can impair the ability of nature [sic] systems to maintain themselves.”
   3. “Wilderness and wildlife are important components in supporting both the physical and spiritual life of humans.”
   4. “Human values go beyond economics to include preservation of wilderness.”
   5. “Existence of wilderness is critical to the well-being of humanity.”
3. *Ecocentrism*
   1. “Natural systems are the basis of all organic existence, including humanity.”
   2. “Human survival is linked to ecosystem survival.”
   3. “Human ethics requires ecologically responsible actions.”
   4. “Proper use of natural sciences can guide the relationship between humanity and its natural environment.”
4. *Political Ecology*
   1. “Domination of humans by other humans leads to domination of nature.”
   2. “The economic system and nation-state are the core structures of society that create ecological problems.”
   3. “Commoditization and market imperatives force consumption to continually increase in the developed economy.”
   4. “Environmental destruction in low-income/racially distinct communities, or Third World countries, originates in the exploitation of the people who live in these areas by the dominant social institutions.”
   5. “Resolution of environmental problems requires fundamental social change based on empowerment of local communities.”
5. *Deep Ecology*
   1. “The richness and diversity of all life on earth have intrinsic value.”
   2. “Human relations to nature endanger the richness and diversity of life.”
   3. “Human life is privileged only to the extent of satisfying vital needs.”
   4. “Maintenance of the diversity of life on earth mandates a decrease in human impacts on the natural environment and substantial increases in the wilderness areas of the globe.”
   5. “Changes affecting basic economic, technological, and cultural aspects of society are required to realize this goal.”
6. *Ecofeminism*
   1. “Earth is home for all life and should be revered and nurtured.”
   2. “Ecosystem abuse is rooted in androcentric concepts and institutions.”
   3. “Relations of complementarity rather than superiority between culture/nature, human/nonhuman, and male/female are desirable.”
   4. “The many problems of human relations, and relations between human and nonhuman worlds, will not be resolved until androcentric institutions, values, and ideology are eradicated.”

Martinez-Alier 2002 [12]:

1. *Cult of the Wilderness*
   1. “... concerned with the preservation of wild Nature but without anything to say on industry and urbanization, indifferent or opposed to economic growth, most worried by population growth, backed up scientifically by conservation biology …” (p. 14)
2. *Gospel of Eco-Efficiency*
   1. “... concerned with the sustainable management or ‘wise use’ of natural resources and with the control of pollution not only in industrial contexts but also in agriculture, fisheries and forestry, resting on a belief in new technologies and the ‘internalization of externalities’ as instruments for ecological modernization, backed up by industrial ecology and environmental economics…” (p. 14)
3. *Environmentalism of the Poor*
   1. “... the environmental justice movement, popular environmentalism, the environmentalism of the poor, livelihood ecology, and liberation ecology, grown out of local, regional, national and global ecological distribution conflicts caused by economic growth and social inequalities. … Actors in such conflicts have often not used an environmental idiom, and this is one reason why this old third current of environmentalism was not identified until the 1980s and 1990s.” (p. 14)

Bäckstrand and Lövbrand 2006 [13]:

1. *Ecological Modernization*
   1. “The distinct feature of ecological modernization is the compatibility of economic growth and environmental protection, a liberal market order and sustainable development” (p. 52)
   2. “in favor of a gradual transformation of the state and market to promote green regulation, technology, investment and trade” (p. 53)
   3. “technocratic greening of industrial production” (p. 53)
2. *Green Governmentality*
   1. “This discourse epitomizes a global form of power tied to the modern administrative state, mega-science and big business. It entails the administration of life itself—individuals, populations and the natural environment” (p. 53-54).
   2. “a notion of stewardship of nature and an all-encompassing management of its resources” (p. 54)
   3. “nature is approached as a terrestrial infrastructure subject to state protection, management and domination” (p. 55)
3. *Civic Environmentalism*
   1. “in order build more effective environmental multilateralism groups who are affected by environmental problems, or have a legitimate interest or stake, should have a voice in finding solutions” (p. 55)
   2. “Attention has been given to the “participation gap” in global environmental politics where the inclusion of so-called marginalized groups (women, youth, indigenous people, etc.) is seen as critical in realizing sustainable development” (p. 55).
   3. “The *reformist civic environmentalism* discourse, which can be conceived of as ‘participatory multilateralism’ stresses how the vital force of a transnational civil society complements state-centric practices. … This reformist discourse promotes a pluralistic global environmental order and affirms the rise of public-private partnerships between NGOs, business and governments as they hold the promise of result-based environmental problem-solving” (p. 56)
   4. “a more *radical edge of the civic environmentalism* discourse is deeply skeptical of the promise of stakeholder governance. Drawing on a neo- Gramscian perspective, this discourse highlights how relations of power and powerlessness are at the core of international institutions and negotiation processes. It is informed by a radical ecology agenda that advocates a fundamental transformation of consumption patterns and existing institutions to realize a more eco-centric and just world order” (p. 56)
      1. “global social movements should challenge and resist inequitable power structures” (p. 56)

**SUPPLEMENTARY REFERENCES**

1. Mitchell T. The limits of the state: beyond statist approaches and their critics. Am Polit Sci Rev. 1991;85: 77–96.

2. MaxQDA. MaxQDA (12.3.5). Berlin, Germany: VERBI Software; 2017.

3. Doak DF, Bakker VJ, Goldstein BE, Hale B. What is the future of conservation? Trends Ecol Evol. 2014;29: 77–81.

4. Abdi H. The Kendall Rank Correlation Coeffiscient. Encycl Meas Stat. 2007;

5. Zhu M, Ghodsi A. Automatic dimensionality selection from the scree plot via the use of profile likelihood. Comput Stat Data Anal. 2006;51: 918–930. doi:10.1016/j.csda.2005.09.010

6. Cattell RB. The Scree Test for the Number of Factors. Multivariate Behav Res. 1966;1: 245–276. doi:http://dx.doi.org/10.1207/s15327906mbr0102_10

7. Johnstone IM. On the Distribution of the Largest Eigenvalue in Principal Components. Ann Stat. 2001;29: 295–327.

8. Micallef L, Rodgers P. euler APE: Drawing area-proportional 3-Venn diagrams using ellipses. PLoS One. 2014;9. doi:10.1371/journal.pone.0101717

9. Herndl C, Brown S, editors. Green Culture: Environmental Rhetoric in Contemporary America. Madison, WI: University of Wisconsin Press; 1996.

10. Dryzek JS. The Politics of the Earth: Environmental Discourses. Oxford: Oxford University Press; 1997.

11. Brulle RJ. Environmental discourse and social movement organizations. Sociol Inq. 1996;66: 58–83.

12. Martínez-Alier J. The Environmentalism of the Poor: A Study of Ecological Conflicts and Valuation. Northampton, MA: Edward Elgar Publishing; 2002.

13. Bäckstrand K, Lövbrand E. Planting Trees to Mitigate Climate Change: Contested Discourses of Ecological Modernization, Green Governmentality and Civic Environmentalism. Glob Environ Polit. 2006;6: 50–75.
